# Heterogeneous human–robot task allocation based on artificial trust

**DOI:** 10.1038/s41598-022-19140-5

**Published:** 2022-09-12

**Authors:** Arsha Ali, Hebert Azevedo-Sa, Dawn M. Tilbury, Lionel P. Robert

**Affiliations:** 1 Robotics Department, University of Michigan, Ann Arbor, MI USA; 2 Military Institute of Engineering, Rio de Janeiro, Brazil; 3 Department of Mechanical Engineering, University of Michigan, Ann Arbor, MI USA; 4 School of Information, University of Michigan, Ann Arbor, MI USA

**Keywords:** Computational science, Computer science

## Abstract

Effective human–robot collaboration requires the appropriate allocation of indivisible tasks between humans and robots. A task allocation method that appropriately makes use of the unique capabilities of each agent (either a human or a robot) can improve team performance. This paper presents a novel task allocation method for heterogeneous human–robot teams based on artificial trust from a robot that can learn agent capabilities over time and allocate both existing and novel tasks. Tasks are allocated to the agent that maximizes the expected total reward. The expected total reward incorporates trust in the agent to successfully execute the task as well as the task reward and cost associated with using that agent for that task. Trust in an agent is computed from an artificial trust model, where trust is assessed along a capability dimension by comparing the belief in agent capabilities with the task requirements. An agent’s capabilities are represented by a belief distribution and learned using stochastic task outcomes. Our task allocation method was simulated for a human–robot dyad. The team total reward of our artificial trust-based task allocation method outperforms other methods both when the human’s capabilities are initially unknown and when the human’s capabilities belief distribution has converged to the human’s actual capabilities. Our task allocation method enables human–robot teams to maximize their joint performance.

## Introduction

Human–robot collaboration involves humans and robots performing tasks together within the same collaborative workspace to achieve overarching goals^1–3^. Human–robot teams can lead to better performance, productivity, reliability, and ergonomics^3,4^. Thus, robots are being introduced across various industries to work with humans. For example, humans and robots can work together to assemble a vehicle in a manufacturing plant or perform a surgery in an operating room. However, collaboration cannot be effective unless each agent is allocated the appropriate tasks. In our work, we consider an agent to be either a human or a robot. As a result, there is a need for effective human–robot task allocation methods.

**Figure 1:**
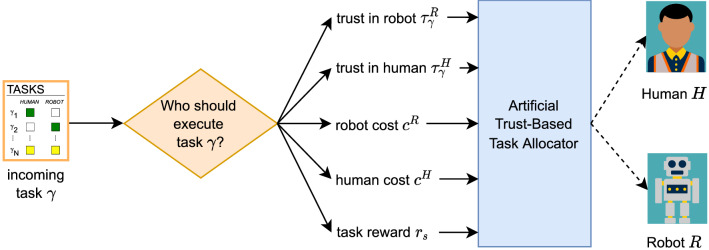
Overview of our artificial trust-based task allocation method. In task allocation, each incoming indivisible task must be allocated to and executed by one agent on the human–robot team. An artificial trust-based task allocation method can be used to allocate tasks by considering trust in each agent from the robot’s perspective, cost of each agent, and task reward.

Effective human–robot collaboration requires the appropriate allocation of indivisible tasks between humans and robots which begs the question: *which agent should do what?* Task allocation answers this question. Task allocation is vital to successful human–robot collaboration, but there are concerns about many existing human–robot task allocation methods that would benefit from further research. First, many existing task allocation methods assume that agent capabilities are known beforehand (e.g., see^5–9^). While agents can come to learn the capabilities of their teammates through interactions, they may not be known initially, especially for newly formed human–robot teams with heterogeneous agents. Second, many task allocation methods are developed for a predefined specific type of task, such as routing^8^, delivery^10^, surveillance^11^, and assembly or manufacturing operations^2,7,9,12–16^. However, robots are being deployed across a variety of settings. This means that human–robot teams can encounter a wide variety of tasks across domains.

In this paper, we propose a human–robot task allocation method that addresses these concerns and extends existing human–robot task allocation methods by incorporating trust in each agent from the robot’s perspective, as well as agent cost and task reward, to allocate indivisible tasks to one agent on the team as shown in Fig. 1. Cost is viewed as the price that needs to be paid to have an agent execute a task, and task reward is viewed as the revenue for successful task completion. As we will discuss in the background and method sections, the model for computing trust does not require known agent capabilities, and allows for allocation of novel tasks by comparing task requirements with the belief in agent capabilities. We simulated our task allocation method for a human–robot dyad and compared its performance with a random task allocation method and the human–robot task allocation method by Tsarouchi *et al.*^9^. We demonstrated how team total reward from our human–robot task allocation method outperforms these other methods.

The research questions we answer are:

- Can a human–robot task allocation method account for both unknown agent capabilities and novel tasks?
- How does the performance of this human–robot task allocation method compare with other methods?

The primary contribution of this work is an artificial trust-based task allocation method for heterogeneous human–robot teams that (i) learns agent capabilities and develops trust in an agent over time, (ii) allocates both existing and novel tasks, and (iii) outperforms other methods in terms of team total reward.

## Background

### Human-automation and human–robot collaboration

Research on robots works to understand how robots can work collaboratively with humans rather than simply replacing humans^17^. Although robots are becoming more advanced, robots still cannot complete all types of tasks that can be performed by humans^18^. In fact, we now know that humans are generalists that perform many types of tasks, while robots excel at narrow and standalone tasks^19^. This makes human–robot teams advantageous because they can combine the strengths of both human and robotic agents^3,4,14^. Research has also shown that people are willing to work with robots^20^, particularly when robots can offer skills that humans lack. For example, Wiese *et al.* found that participants collaborated with robots especially when the participant’s own capabilities for the task were low and the robot seemed more capable^21^.

Humans and automation can collaborate together at different levels to improve performance and reduce human burden. In human-automation teaming, Sheridan and Verplank proposed 10 levels for decision-making ranging from level 1 where there is no automation to level 10 where there is full automation^22^. Also, Parasuraman, Sheridan, and Wickens developed a model that included the application of automation to four functions (information acquisition, information analysis, decision and action selection, action implementation), each of which can range from no automation to full automation^23^. In contrast to fixed automation, flexible automation is when the level and/or type of automation can vary during system operations^24^. Adaptable automation is when a change in the level of automation is initiated by a human, whereas adaptive automation is when automation makes such changes^24,25^. A recent long-term study made use of an adaptive collision avoidance system where the type of steering automation was selected based on the location of a vehicle in the adjacent lane^26^.

Inspired by such levels of automation, levels of collaboration have been proposed for human–robot teams, ranging from no coexistence to full collaboration^27^. One robot designed to operate with humans in close proximity is the Baxter robot, with a set of eyes as a way to communicate with a human^28^. Similar to human-automation teaming, agents can take on different roles and responsibilities in human–robot teaming. In line with the effort to reduce human cognitive burden, in our work, tasks are allocated by a robot so as not to overburden a human by being responsible for both delegating and executing tasks. In addition, one study found that total completion time was reduced by 10% when task allocation was done by a robot instead of task allocation done by a human^29^, which further motivates tasks being allocated by a robot.

### Prior task allocation methods

Generally, multi-robot and human–robot task allocation methods can be classified into three types: homogeneous agent-based, capabilities-based, and capacity-based. Homogeneous agent task allocation is typically undertaken in structured environments, where all of the agents and tasks are of the same type and any agent can perform any task^8,10^. Homogeneous task allocation is based on the assumption that all agents and performances across agents are identical, which is why these methods are usually applicable to multi-robot teams and not human–robot teams. For example, Jeon, Lee, and Kim select a robot specifically for a hospital delivery task based on traveling distance^10^.

Capability-based task allocation methods consider the heterogeneity of agents, commonly seeking to match the capabilities or types of agents with task demands^2,5–7,9,18^. Heterogeneous agents vary in their capabilities, operating areas, and communication capabilities^6^. Fitts list or MABA-MABA (men-are-better-at, machines-are-better-at) is known as a classical theory outlining the general strengths of humans and machines^30^ and has been used as a basis for function allocation^31^. An example of a capability-based task allocation method is one by Tsarouchi *et al.*, which uses a set of decision steps, allocating a task to a capable and available agent that can execute the task with the minimum operation time^9^.

Capacity-based (or adaptive automation) methods rely on human capacity information (e.g., workload, fatigue) to aid in the allocation of tasks (or level of automation control), aiming to keep capacity in acceptable ranges^11,12,32–35^. These methods may also use information about the capabilities of agents, current performance, environment, or context^24–26^. For example, Hu and Chen use a continuous-time Markov decision process (MDP) to model human fatigue as a measure of human capacity when allocating tasks^12^.

Incorporating trust in task allocation has been introduced before for contractor/auctioneer agents^36^ and distributed systems^37^. In the law enforcement domain, task allocation has also considered tasks whose location, arrival time, and importance is unknown a priori^38^. Unlike these methods, our task allocation method incorporating trust is specifically for human–robot teams. Although Jiang briefly mentions task allocation in distributed systems can be based on trust^37^, there is no specific discussion of how trust can be used to improve performance. Dash, Ramchurn, and Jennings’s method requests subjective inputs and trust functions from agents before the allocation of tasks^36^. However, in dynamic situations, time-critical decisions may need to be made and there may not be time to gather and process input from multiple agents. Our method, as we explain in the following sections, concretely formalizes a trust model and allocates tasks without requesting input from other agents on the team. While Tkach and Amador’s method deals with a similar problem as us in that tasks are not known in advance but in a specific domain^38^, there is no discussion of how to account for agent skills that are unknown nor of how trust may evolve between agents. Our method learns unknown capabilities of an agent over time using stochastic task outcomes, which then impacts the evolution of trust in that agent. While task allocation has been studied in different contexts, we are specifically interested in task allocation for human–robot teams.

### Trust definition and dimensions

In this paper, we provide an answer to the questions of how agent capabilities can be learned when they are not known in advance and how novel tasks can be allocated in a task allocation method. Our human–robot task allocation method aims to build upon existing methods through our formulation of trust as an element in the task allocation method. Although there are many definitions of trust (e.g., see^39,40^), a recent paper found common ground that trust is “a dyadic relation in which one person accepts vulnerability because they expect that the other person’s future action will be governed by certain characteristics”^41^. Hence, in our work, trust is defined as the trustor’s (the agent who trusts) willingness to be vulnerable to the trustee’s (the agent who is trusted) actions^42,43^ and represented as the probability that a given agent will successfully execute a given task. Trust is a multi-dimensional construct and can be influenced by, for example, capability (skills, knowledge, competence), reliability (consistency or predictability), honesty (being truthful), benevolence (good intentions), and integrity (following moral principles)^41,42,44^. All trust dimensions can influence the probability that an agent will successfully execute a task. Trust can also be influenced by physical characteristics such as human-likeness of a robot^45^, the type of agent and context^46^, and institutional perspectives^47^. In this paper, we simplify the trust estimate by considering only the dimension of capability, since trust in automation primarily focuses on performance^41^ and robot performance is an important and strong contributor to trust in human–robot interaction (HRI)^48,49^.

Trust is an important concept present in human-automation teams and is also needed in human–robot teams for effective collaboration^50,51^. When trust is miscalibrated (meaning trust is not aligned with the agent’s capabilities), the trustor can overtrust or undertrust the trustee. Overtrust can lead to misuse, where the trustor relies on the trustee to execute tasks beyond the trustee’s capabilities, and undertrust can lead to disuse, where the trustor does not fully leverage the capabilities the trustee offers^43,52^. Miscalibrated trust can result in suboptimal outcomes, motivating the development of trust-aware robots that can modify their behavior to manipulate or repair humans’ trust^53–55^. Many trust models and real-time trust measures exist in the literature (e.g., see^56–59^). These are not discussed further in this paper since our focus is on developing a novel human–robot task allocation method based on an existing trust model^60^, although our task allocation method can also be used with other trust models that estimate trust numerically.

### Artificial trust model (ATM)

Consider a general scenario of many tasks arriving with their required levels of capabilities. These tasks could be anything (e.g., sorting, search and rescue) relevant to the domain of the human–robot team. Each agent on the team has a proficiency level for each capability dimension, which are labels that describe distinct skills (e.g., sensing, processing, speech, navigation). Agents are heterogeneous, meaning each agent can be good at different tasks (i.e., have different capabilities). We envision a standard set of capability dimensions depending on the operational domain of the human–robot team. With newly formed teams, agents do not have a good idea of which teammates can be trusted with which tasks, but will develop trust in a trustee over time. These ideas are used in our trust model^60^.

The trust model^60^ predicts both *natural* and *artificial* trust. Natural trust is human trust in another agent, and artificial trust is robotic trust in another agent. In this paper, we focus on artificial trust. The *n* distinct capability dimensions are represented as a capability hypercube by the Cartesian product $\Lambda = \prod _{i=1}^n \Lambda _i = [0, 1]^n$. A task $\gamma \in \Gamma$ is represented by its required capabilities $\bar{\lambda } = (\bar{\lambda }_1, \bar{\lambda }_2, ..., \bar{\lambda }_n) \in \Lambda$, and every agent’s capabilities $\lambda ^a = (\lambda _1^a, \lambda _2^a, ..., \lambda _n^a) \in \Lambda$ are represented by a capabilities belief distribution $bel(\lambda ^a) = (\ell ^a, u^a)$, where *a* is one specific agent on the team *T*. We use the terms agent’s capabilities and agent’s actual capabilities interchangeably in this paper, but this is different from an agent’s capabilities belief distribution. The capabilities belief distribution is always a uniform distribution with a lower bound of $\ell ^a = (\ell _1^a, \ell _2^a, ..., \ell _n^a) \in \Lambda$ and an upper bound of $u^a = (u_1^a, u_2^a, ..., u_n^a) \in \Lambda$. Belief distributions are initialized as uniform with a lower bound of 0 and an upper bound of 1 for each capability dimension $\Lambda _i$. Trust in an agent to successfully complete a task is higher when the agent’s capabilities belief exceeds the task requirements. A trustee’s capabilities belief is developed over time, as the trustee agent is observed either succeeding or failing at tasks.

In the *ATM*, the robot keeps a history of performances and computes and updates trust as follows. The robot’s trust in another agent *a* (either a human or a robot) to execute task $\gamma$ at time *t* is given by $\tau _\gamma ^a$ in Eq. (1), where $\psi (\bar{\lambda }_i)$ is given by Eq. (2).

1 $$\begin{aligned} \tau _\gamma ^a(a, \gamma , t) & = \prod _{i=1}^n \psi (\bar{\lambda }_i) \end{aligned}$$

2 $$\begin{aligned} \psi (\bar{\lambda }_i) & = \left\{ \begin{array}{ll} 1 & \text{ if } 0 \le \bar{\lambda }_i \le \ell _i^a, \\ \frac{u_i^a - \bar{\lambda }_i}{u_i^a - \ell _i^a} & \text{ if } \ell _i^a< \bar{\lambda }_i < u_i^a, \\ 0 & \text{ if } u_i^a \le \bar{\lambda }_i \le 1 \end{array} \right. \end{aligned}$$

The product of probabilities is taken because capability dimensions are considered to be independent, which will require careful selection of the capability dimensions in practice. If capability dimensions are interrelated, such as sensing and identification could be, they can be combined into one dimension. Trust for each capability dimension $i \in \mathbb {N}^+, i \in [1, n]$ is computed by considering the lower and upper bounds $(\ell _i^a, u_i^a)$ of the capability belief with the task requirement $\bar{\lambda }_i$ in that dimension. If the task requirement $\bar{\lambda }_i$ is less than or equal to the capability belief lower bound $\ell _i^a$ (i.e., $\bar{\lambda }_i \le \ell _i^a$), trust for that capability dimension is 1. On the other hand, if the task requirement $\bar{\lambda }_i$ is greater than or equal to the capability belief upper bound $u_i^a$ (i.e., $\bar{\lambda }_i \ge u_i^a$), trust for that capability dimension is 0. When the task requirement $\bar{\lambda }_i$ falls between the lower and upper bounds of the trustee agent’s capability belief (i.e., $\ell _i^a< \bar{\lambda }_i < u_i^a$), trust decreases linearly from 1 to 0. In this paper, trust is not synonymous with capability. Trust is a result of the comparison between the belief in the agent’s capabilities and the task requirements, as opposed to knowing the agent’s actual capabilities. By comparing continuous task requirements with the belief in agent capabilities, this model can predict trust on novel tasks the human–robot team has not seen before.

To update the capabilities belief distribution $bel(\lambda ^a)$, an optimization problem is solved. After observing the outcome of the task execution, trust is approximated as $\hat{\tau }_\gamma ^a$ by the number of task successes divided by the total number of times task $\gamma$ was executed up to the current time *t* by the agent *a* as given in Eq. (3). The outcome of task $\gamma$ being executed by agent *a* at time *t* is given by $\Omega (a, \gamma , t) \in \{0,1\}$. The outcome 0 is a failure and 1 is a success. The complement of $\Omega$ is given by $\mho$, which assigns a 1 for failure and 0 for success.

3 $$\hat{\tau}_\gamma^a = \frac{\sum\limits_{m=0}^t \Omega(a, \gamma, m)}{\sum\limits_{m=0}^t \big[\Omega(a, \gamma, m)
+ \mho(a, \gamma, m)\big]}$$

The capabilities belief distribution lower and upper bounds $(\ell ^a, u^a)$ are recursively updated to $(\hat{\ell }^a, \hat{u}^a)$ as given in Eq. (4) to minimize the difference between the trust approximation $\hat{\tau }_\gamma ^a$ based on task outcomes and trust ${\tau }_\gamma ^a$ computed by the *ATM*. The capability hypercube $\Lambda$ can be discretized for numerical computations.

4 $$\begin{aligned} \begin{aligned} (\hat{\ell }^a, \hat{u}^a) = \mathop {\mathrm{arg}\,\mathrm{min}}\limits _{[0, 1]^n} \int _{\Lambda } \Vert \tau _\gamma ^a - \hat{\tau }_\gamma ^a \Vert ^2 d\lambda \end{aligned} \end{aligned}$$

Our prior work proposed a bi-directional trust model^60^ and the idea of using this bi-directional trust model for task allocation, without any results^61^. In this paper we develop the details needed to apply the artificial trust model to a human–robot task allocation method, apply it to a scenario with two capability dimensions, run the simulations, and present and interpret the results.

## Artificial trust-based task allocation method development

### Overview and characteristics of our task allocation method

Existing human–robot task allocation methods provide insight as to how agents can best work together as a unified team. However, there is an opportunity to further investigate human–robot task allocation in cases where agents’ capabilities and tasks are not known in advance. In our work, we consider a task allocation problem of both existing and novel tasks arriving at unknown times. This is different from task scheduling problems in which a set of tasks is known in advance such that they can be sequenced (e.g., see^62–64^). According to Korsah *et al.*’s taxonomy for multi-robot task allocation^65^ (which includes Gerkey and Matarić’s taxonomy^66^), our problem falls in the no dependencies (ND) category, and as single-task (ST), single robot (SR) (where we generalize robot to mean agent in our case), and instantaneous assignment (IA) (ND[ST-SR-IA]). Although some tasks do have dependencies, there are also tasks without dependencies to which our task allocation method is applicable. After a task is allocated to an agent, we envision the agent will execute the task as soon as they are available and before another task is allocated to them. Thus, in this paper, we focus on which agent should do what task and not on when they should do it.

**Figure 2:**
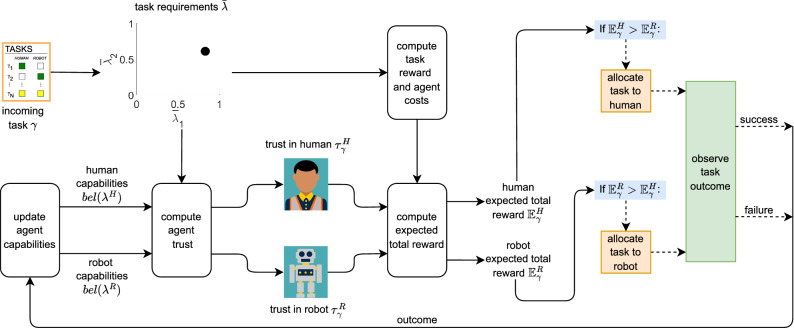
Flowchart with the main ideas of our artificial trust-based task allocation method for a team consisting of one human and one robotic agent. The process starts with an incoming task (black dot) defined by a set of task capability requirements. In this case, the incoming task is defined by two capability dimensions. The trust in each agent is computed using the capabilities belief distribution of that agent. The task reward and agent costs are computed using the task requirements. The expected total reward for each agent is computed using trust in the agent, task reward, and agent cost. The agent that maximizes the expected total reward is allocated the task. The outcome of the task is observed as a success or a failure, which is used to update the capabilities belief distribution of the agent that executed the task. The process continues for each incoming task.

In our method, following the ATM, tasks are represented by the levels of capabilities required to successfully execute the task, and agents are represented by the levels of capabilities they possess. Since human trust in a robot is known to transfer across different tasks^67^, trust in an agent to execute a new task can be reasoned about by considering the similarity between the new task capability requirements with existing tasks. Thus, our method is able to allocate any task that can be represented by standard capability dimensions. Also, our method does not assume that the capabilities of an agent are known beforehand. The belief in an agent’s capabilities is updated over time as task outcomes are observed, either as successes or as failures. Task outcomes are not assumed to be strictly successes or failures, making them stochastic.

We also consider the reward associated with the task and the cost of using each agent, thereby considering the trade off between trust in the agent and cost of the agent. Task allocation is done using the robot’s opinion on who should execute the task. The observer of the human–robot team is the robot (or can be a third-party agent depending on the HRI domain). We implemented our task allocation method through a simulation of a human–robot dyad, showing the allocation of tasks with different capability requirements and measuring performance and team total reward. The results demonstrated the benefits of our human–robot task allocation method in comparison to other methods.

### Artificial trust-based task allocation (ATTA) Method

To allocate a task, the ATTA method uses trust as computed from the ATM, along with the cost of using a specific agent to do the task and the reward for successfully executing the task. The penalty for failing at the task can also be included in the method if desired. Trust in the agent, task reward, and agent cost are used to calculate the expected total reward for each agent for a given task, and the task is then allocated to the agent that maximizes the expected total reward function. The expected total reward can be thought of as expected profit, where profit is revenue minus cost.

Figure 2 and Supplementary Algorithm S1 describe the ATTA method for a team of one human and one robotic agent, although our method can scale to larger human–robot teams. An agent is represented by $a \in T = \{H,R\}$, where *H* represents the human agent and *R* represents the robotic agent. Each indivisible incoming task $\gamma \in \Gamma$, represented by a set of capability requirements $\bar{\lambda }$, needs to be allocated to one agent on the team. $\Gamma$ is a set that updates to hold incoming tasks that are not yet allocated. Using the current belief in the human’s capabilities $bel(\lambda ^H)$ and the robot’s capabilities $bel(\lambda ^R)$ and the task requirements $\bar{\lambda }$, trust in the human $\tau _{\gamma }^{H}$ and trust in the robot $\tau _{\gamma }^{R}$ are computed following Eqs. (1) and (2). In our ATTA method, trust in an agent is evaluated from the robotic agent’s perspective. Since the robotic agent is also computing self-trust, we assume that the robot is aware of its own capabilities (i.e., the robot’s capabilities are known and $bel(\lambda ^R) = \delta (\lambda - \lambda ^R)$, meaning $\ell ^R = \lambda ^R$ and $u^R = \lambda ^R$).

Next, the task requirements $\bar{\lambda }$ are used to compute the task reward $r_s$ (revenue) and agent costs $c^H$ and $c^R$ (cost) for both the human and robotic agents. The expected total reward (expected profit) can now be calculated for each agent $\mathbb {E}_\gamma ^H$ and $\mathbb {E}_\gamma ^R$, which depends on the trust in the agent, task reward, and agent cost. If the expected total reward of both agents falls within a tolerance $\alpha$, the task is allocated to the agent with fewer tasks already allocated to it, where $k^H$ and $k^R$ are the number of tasks already allocated to the human and robot respectively. Otherwise, the task is allocated to the agent that maximizes the expected total reward.

Equation (5) gives the expected total reward equation $\mathbb {E}_\gamma ^a[total\;reward]$ for an agent *a*,

5 $$\begin{aligned} \mathbb {E}_\gamma ^a[total\;reward] = {\tau }_\gamma ^a(r_{s}-c^a) + (1-{\tau }_\gamma ^a)(r_{f}-c^a) , \end{aligned}$$

where trust ${\tau }_\gamma ^a$ is the probability of agent *a* successfully executing the task $\gamma$ and $1-{\tau }_\gamma ^a$ is the probability of failing as outputted by the ATM. The reward for task success is given by $r_s$ and the penalty for failing the task is given by $r_f$. The cost of using agent *a* to execute the task is given by $c^a$.

Assuming there is no penalty for task failure (i.e., $r_f = 0$), Eq. (5) reduces to Eq. (6),

6 $$\begin{aligned} \mathbb {E}_\gamma ^a[total\;reward] = {\tau }_\gamma ^ar_{s}-c^a . \end{aligned}$$

In practice, this assumption should be made cautiously as $r_f$ can be important, especially when there is potential for human injury or loss of life. The simulations run in this paper do not include a penalty for task failure, but a penalty can be included in our ATTA method if appropriate and desired.

Assuming that task $\gamma$ complexities are fully described by the task requirements $\bar{\lambda }$, the reward for success $r_s$ function can depend on the task requirements $\bar{\lambda }$ (i.e., $r_s = f_r(\bar{\lambda })$, $\bar{\lambda } \in [0, 1]^n$). The cost of an agent to execute a task $c^a$ can depend on the specific agent, either a human or a robot in this case, and the task requirements (i.e., $c^a = f_c(a, \bar{\lambda })$, $a \in T = \{{H, R}\}$).

To contextualize $r_s$ and $c^a$, consider sorting tasks where items need to be classified and transported to their correct locations. These tasks can be represented by capability dimensions of classification and manipulation. Being able to classify an item has no influence on being able to manipulate it, and vice versa. Hence, classification and manipulation are independent capability dimensions. An item that is very distinct from all others and has a lighter weight will be easier to classify and manipulate than an item that could be mistaken for another and has a heavier weight. For any agent, the former item would have a lower $r_s$ and $c^a$ than the latter item.

After an agent executes a task, the task outcome is observed by the robot (or third-party agent when applicable) as either a success or a failure. For the simulation, the task outcome is determined using true trust $\bar{\tau }_\gamma ^H$ in the human and true trust $\bar{\tau }_\gamma ^R$ in the robot, which is computed using the actual capabilities of each agent $\lambda ^a$ and the uncertainty in task execution $\eta$ in Eq. (7), as opposed to predicted trust $\tau _\gamma ^H$ and $\tau _\gamma ^R$ which was used for task allocation. The probability of the task outcome being a success for task $\gamma$ when executed by agent *a* is the true trust probability given by $\bar{\tau }_\gamma ^a$ and the probability of the task outcome being a failure is given by $1- \bar{\tau }_\gamma ^a$. The agent’s actual capabilities predict the task outcome because the success of the task will rely on the actual capabilities of the agent, not on the belief in capabilities. $\eta$ is a factor that captures uncertainty in the execution of the task (e.g., due to workload, fatigue, or environmental noise). Each capability dimension $\Lambda _i$ can have a different uncertainty parameter $\eta _i$, or there can be one scalar $\eta$ used for all capability dimensions. As ${\eta _i\rightarrow \infty }$, there is greater uncertainty and we approach a true trust of 0.5, meaning that any task could be either a success or a failure regardless of the task requirements and the agent’s actual capabilities. True trust is computed only for the simulation. In practice, the task outcome can be readily observed so Eq. (7) would not be computed.

7 $$\begin{aligned} \begin{aligned} \bar{\tau }_\gamma ^a = \prod _{i=1}^n \frac{1}{1 + e^{\frac{ (\bar{\lambda }_i - \lambda _i^a)}{\eta _i}}} \end{aligned} \end{aligned}$$

Finally, if the task was executed by the human, the human’s capabilities belief $bel(\lambda ^H)$ is updated using the capability update procedure of the ATM in Eqs. (3) and (4). The process repeats for each incoming task that has to be allocated.

## Simulation

### General setup

We tested our ATTA method in a simulation environment with a team of one human and one robotic agent who have different $\lambda _1^a$ and $\lambda _2^a$ capability values for unspecified $\Lambda _1$ and $\Lambda _2$ capability dimensions as listed in Table 1. The human’s capabilities and the robot’s capabilities were chosen to show a clear division in the capability hypercube where tasks are being allocated to the human and where tasks are being allocated to the robot, which is presented with the results. Neither the human nor the robot was given full capability in either capability dimension to highlight that tasks that no agent is capable of may still arise and will have to be allocated. The capability in each dimension is different for each agent and between agents to emphasize that agents can have different proficiencies in different capability dimensions and that agents are heterogeneous. Considering the sorting example again, a robot may be able to manipulate heavier items but have difficulty with classification, whereas a human may be better at classifying items but can only manipulate lighter items. Thus, the capability values chosen reflect how humans and robots have different strengths and why human–robot teaming is advantageous. We chose to leave the capability dimensions unspecified since our method is not limited to a particular HRI domain. These same capability dimensions and agent capability values are used in the task allocation methods we compare against ATTA. If these agent capability values were known for a real situation, the implementations and comparisons would be similar. In these simulations, the robot does the task allocation.

The simulation was run 10 times for the allocation of $N=500$ unspecified tasks using Python 3.8.3. Based upon power analysis, we decided to run 10 simulations to demonstrate statistical significance in our results. We chose to allocate $N=500$ tasks because this aids in showing a clear division of which tasks are allocated to which agent in the capability hypercube $\Lambda$. Each task’s $\bar{\lambda }_1$ and $\bar{\lambda }_2$ capability requirement was sampled from the probability density functions in Supplementary Fig. S1, reflecting that lower requirement tasks are more frequent than high requirement tasks. Histograms for one sample of $N = 500$ tasks are overlaid in Supplementary Fig. S1.

The task reward function $r_s \in [0, 1]$ was defined as the average of the task requirements as $r_{s} = \frac{1}{2}(\bar{\lambda }_1 + \bar{\lambda }_2)$. The robot cost function $c^R \in [0, 0.25]$ and the human cost function $c^H \in [0, 0.667]$ were defined as a weighted linear combination of the task requirements as $c^R = \frac{1}{8}(\bar{\lambda }_1 + \bar{\lambda }_2)$ and as $c^H = \frac{1}{3}(\bar{\lambda }_1 + \bar{\lambda }_2)$ respectively. The reward for success $r_s$, robot cost $c^R$, and human cost $c^H$ indicate that each capability dimension is equally as important when executing a task. When creating the human cost and robot cost functions, we followed two requirements. First, the human and robot costs for a task are designed to be less than the task reward for success. Second, the robot cost was chosen to be less than the human cost for any given task requirement because it is a well established fact that robots can reduce costs as compared to humans^68^. The human and robot cost functions also indicate that both agent costs are not negligible, and therefore are important. In this version, we consider a tolerance of $\alpha = 0$, where the task is allocated to the agent that maximizes the expected total reward or to the agent with fewer tasks when the expected total reward between agents is equal.

The outcome of each task, either a success or a failure, was determined by computing true trust $\bar{\tau }_\gamma ^a$ using each agent’s actual capabilities with $\eta _i = \frac{1}{50}, i \in \{1, 2\}$ in Eq. (7), instead of the capabilities belief $bel(\lambda ^H)$ for the human. Thus, as the task outcome is dependent on a probability of true trust $\bar{\tau }_\gamma ^a$, the task outcome is not deterministic. The value of $\eta _i, i \in \{1, 2\}$ was set to $\frac{1}{50}$ because it gave uncertainty in task outcomes near an agent’s actual capabilities, but not in tasks further away.

**Table 1:** Human and robot capabilities for case I (converged or accurate human capabilities) and case II (unconverged or inaccurate human capabilities).

Case	Agent	$\lambda _1^a$ Capability	$\lambda _2^a$ Capability
I	Human	0.55	0.75
	Robot	0.7	0.4

Case	Method	$\lambda _1^H$ Capability	$\lambda _2^H$ Capability
II	ATTA	$(\ell _1^H, u_1^H)$	$(\ell _2^H, u_2^H)$
	Tsarouchi *et al.*^9^ $({+0.1})$	0.65	0.85

### Comparison task allocation methods

Our ATTA method was tested against a random method and the human–robot task allocation method by Tsarouchi *et al.*^9^ under two cases. We decided to test against a random method because it is a commonly used task allocation method. We also decided to test against the method by Tsarouchi *et al.*^9^ because it was the closest comparison method we found to our proposed task allocation method, the applications are similar, and many additional assumptions were not required. The random method and the method by Tsarouchi *et al.*^9^ were close comparison methods because like our method, they can also be applied to allocate indivisible tasks arriving at uncertain times, tasks without dependencies, and both existing and novel tasks. In case I, we allocate tasks after converging on the human’s capabilities given in Table 1 for ATTA and compare with random and with Tsarouchi *et al.*^9^ when the human’s capabilities are accurately known. In case II, we allocate tasks starting from a uniform capabilities belief distribution for the human for ATTA and compare with human capabilities that are inaccurately known by $+0.1$ from the human’s actual capabilities for Tsarouchi *et al.*^9^. Table 1 shows the inaccurate human capabilities that are used in Tsarouchi *et al.*^9^ for comparison against our ATTA method where $(\ell _i^H, u_i^H), i \in \{1,2\}$ are initialized as a uniform distribution with $\ell _i^H = 0$ and $u_i^H = 1$. Tsarouchi *et al.*^9^ $({+0.1})$ means that 0.1 is added to both the human’s $\lambda _1^H$ and $\lambda _2^H$ actual capabilities from case I and then used in the task allocation method by Tsarouchi *et al.*^9^. Although we have not come across other studies with overestimated or underestimated human capabilities, it is possible that human capabilities could be inaccurately known because humans can have a poor estimate of their own and others’ capabilities^69^. Since the random method randomly allocates tasks between the human and the robot without using the agents’ capabilities, there is no difference in the random method between the two cases, so we only compared against the random method in case I.

### Task allocation methods implementation

For determining approximated trust in Eq. (3) and updating the human’s capabilities belief distribution in Eq. (4) for ATTA, for numerical computations, we discretized each capability dimension into 25 equal parts, giving 625 bins for $\Lambda$. We used the mean squared error between the trust approximation based on task outcomes and trust computed by the ATM as the loss function to be minimized in Eq. (4). For this optimization, we used PyTorch^70^ with the Adam algorithm^71^.

For implementing the method by Tsarouchi *et al.*^9^ as a series of decision steps, we considered both the human and robot available to accept a task, agents are capable of executing a task when the task requirements fall within their actual capabilities and incapable otherwise, and agent cost as a proxy for the agent operation time. Tsarouchi *et al.*’s^9^ method is general enough to consider one human and multiple robots, so we found it applicable to our situation with a team of one human and one robot. Since Tsarouchi *et al.*^9^ implies the end of decision making when no agent is capable of the task, we discarded these tasks and did not allocate them. Each discarded task was counted as a failure and we randomly chose whether to count it as a human failure or a robot failure. Tsarouchi *et al.*’s^9^ framework consists of their human–robot task allocation method followed by their scheduling algorithm. Since our ATTA method is not focused on task scheduling but rather on task allocation, we only implemented their task allocation method for comparison and did not use their task scheduling algorithm. Additionally, while Tsarouchi *et al.*^9^ is focused on hybrid assembly cells, we found the task allocation decision steps to be non-exclusive to a particular domain and therefore valid for comparison against ATTA.

## Results

### Comparison metrics

The methods were compared using the metrics of team performance, individual agent performance, and team total reward. All significance testing was done using the Wilcoxon signed-rank test compared to ATTA for the given case using IBM SPSS Statistics 26. The Wilcoxon signed-rank test was chosen as the non-parametric equivalent to the paired sample t-test since normality in our results cannot be guaranteed. The tests were one-tailed. The team performance was calculated as the total number of successfully executed tasks divided by the total number of tasks, in this case, $N = 500$. The performance of each agent was calculated as the number of tasks successfully executed by that agent divided by the number of tasks executed by (or discarded to) that agent. The team total reward was calculated as the summed total reward obtained divided by the summed maximum total reward possible had each task been a success. The total reward for a task is the difference between the task reward (revenue) and cost of the agent that executed the task for task successes, and is a negative cost for task failures (whether the task was executed or discarded) as shown in Eq. (8).

8 $$\begin{aligned} \begin{aligned} total\;reward = \left\{ \begin{array}{ll} r_{s}-c^a & \text{ if } \Omega = 1, \\ -c^a & \text{ if } \Omega = 0 \end{array} \right. \end{aligned} \end{aligned}$$

**Figure 3:**
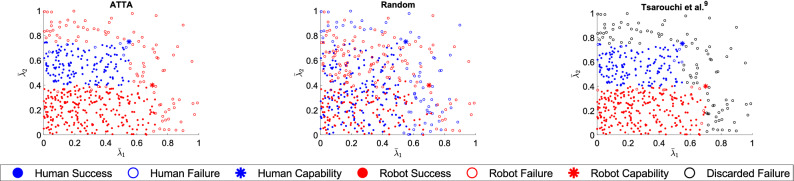
Allocations and outcomes for one sample set of tasks for case I (converged or accurate human capabilities). The outcome, either a success (filled circle) or a failure (unfilled circle), for each task from one sample of $N=500$ tasks as executed by the human (blue) or robot (red) for the ATTA, random, and Tsarouchi *et al.*^9^ methods under case I (converged or accurate human capabilities) is shown. Discarded tasks (black unfilled circle) are failures in Tsarouchi *et al.*^9^. The human’s actual capabilities $\lambda ^H$ (blue asterisk) and the robot’s capabilities $\lambda ^R$ (red asterisk) are also shown.

**Figure 4:**
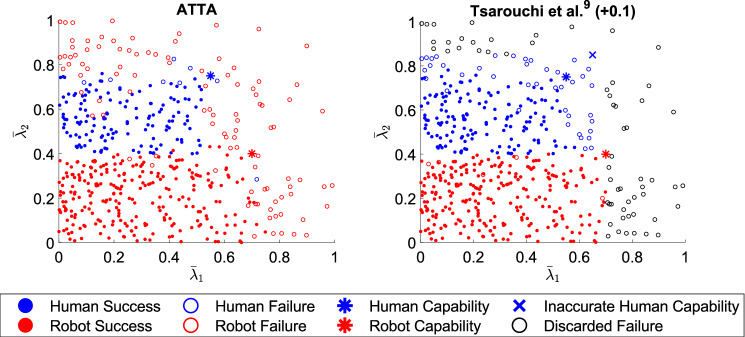
Allocations and outcomes for one sample set of tasks for case II (unconverged or inaccurate human capabilities). The outcome, either a success (filled circle) or a failure (unfilled circle), for each task from one sample of $N=500$ tasks as executed by the human (blue) or robot (red) for the ATTA and Tsarouchi *et al.*^9^ $(+0.1)$ methods under case II (unconverged or inaccurate human capabilities) is shown. Discarded tasks (black unfilled circle) are failures in Tsarouchi *et al.*^9^ $(+0.1)$. Unconverged human capabilities are for ATTA and inaccurate human capabilities (blue cross) are for Tsarouchi *et al.*^9^ $(+0.1)$. The human’s actual capabilities $\lambda ^H$ (blue asterisk) and the robot’s capabilities $\lambda ^R$ (red asterisk) are also shown.

### Case I: Comparison

In case I, our ATTA method was implemented assuming that the human’s capabilities belief has converged to the human’s actual capabilities in Table 1 and there is no need to update the human’s capabilities belief distribution. This was compared against a random task allocation method which does not use agent capabilies to allocate tasks, and against Tsarouchi *et al.*^9^ when the human’s capabilities were accurately known. The allocation and outcomes of tasks for one sample from ATTA, random, and Tsarouchi *et al.*^9^ for case I (converged or accurate human capabilities) are shown in Fig. 3. The team performance, each agent’s performance, and team total reward are shown in Table 2. All comparison metrics were significantly better in ATTA compared to tasks being allocated randomly (all $p = 0.001$). This was expected since the random method does not use agent capabilities when allocating tasks, leaving the allocation completely to chance.

There was no significant difference between our ATTA method and Tsarouchi *et al.*^9^ in terms of team performance ($p = 0.059$). Human performance ($p = 0.001$) and team total reward ($p = 0.001$) were significantly better in ATTA than in Tsarouchi *et al.*^9^, while robot performance was significantly worse ($p = 0.001$). As shown in Fig. 3, the allocation of tasks in ATTA and non-discarded tasks in Tsarouchi *et al.*^9^ was similar, and there were a similar number of successes and failures in these methods, which explains why there was no significant difference in team performance. However, in ATTA, human performance was better and robot performance was worse because most tasks that neither agent was capable of were allocated to the robot, and these were mostly seen as failures. In Tsarouchi *et al.*^9^, the tasks beyond both agents’ capabilities were discarded and randomly chosen as either a human or robot failure. Due to this, the team total reward in ATTA was also better than in Tsarouchi *et al.*^9^ because there were fewer tasks counted as human failures, which are more costly than robot failures.

### Case II: Comparison

In case II, our ATTA method was implemented starting from a uniform capabilities belief distribution for the human, and the human’s capabilities belief distribution was updated after observing the outcome of every task executed by the human. This was compared against Tsarouchi *et al.*^9^ $(+0.1)$ when the human’s capabilities were inaccurately thought to be 0.1 greater than the human’s actual capabilities. The allocation and outcomes for the same sample from ATTA and Tsarouchi *et al.*^9^ $(+0.1)$ for case II (unconverged or inaccurate human capabilities) are shown in Fig. 4. The team performance, each agent’s performance, and team total reward are shown in Table 2. As seen by Fig. 4, in the ATTA method, tasks that fell within both the human and robot’s capabilities were mostly allocated to the robot. This is because the algorithm capitalized on the robot’s low cost and higher trust while the human’s capabilities belief was being learned. Most of these tasks executed by the robot were observed as successes. Most of the tasks beyond the robot’s capabilities but within the human’s capabilities were allocated to the human since trust in the human is higher than trust in the robot, and most of these tasks executed by the human were also observed as successes. Tasks beyond both agents’ capabilities were mostly allocated to the robot, since trust in both agents is low but the robot’s cost is lower, and these tasks were mostly failures. This rule for allocation emerged as the human’s capabilities belief converged, and this is also clearly depicted in Fig. 3 for ATTA after convergence. This rule can be used for future task allocations with these chosen parameters.

For case II in Tsarouchi *et al.*^9^ $(+0.1)$, we saw a similar pattern for task allocation but with respect to inaccurate human capabilities. In Tsarouchi *et al.*^9^ $(+0.1)$, team performance ($p = 0.002$) and robot performance ($p = 0.001$) were significantly better than in ATTA, while human performance ($p = 0.001$) and team total reward ($p = 0.032$) were significantly better in ATTA. When the human was inaccurately thought to be more capable than they actually are in Tsarouchi *et al.*^9^ $(+0.1)$, tasks that should have been discarded were allocated to the human, and these tasks were mostly observed as human failures. This resulted in fewer robot failures (since these tasks were not discarded and could not have been randomly chosen to be robot failures), but at the expense of more human failures. The improvement in team performance over ATTA was due to the elimination of failures that resulted from learning the human’s capabilities. In ATTA, there were additional task failures than in Tsarouchi *et al.*^9^ $(+0.1)$ when the robot was allocated tasks inside the human’s capabilities because the human’s capabilities belief had not yet converged. The significantly worse team total reward in Tsarouchi *et al.*^9^ $(+0.1)$ was due to higher cost human failures both from using inaccurate human capabilities for allocation and failures randomly attributed to the human for discarded tasks, along with failures attributed to the robot for discarded tasks, which outweighed the more, but lower cost, robot failures in ATTA. A similar result emerged with human capabilities 0.1 below the human’s actual capabilities in Tsarouchi *et al.*^9^ $(-0.1)$, where now tasks that the human was capable of were discarded, making team performance significantly worse than in ATTA. Thus, there can be a benefit to team performance and team total reward when learning a trustee’s capabilities rather than using underestimated or overestimated inaccurate capabilities.

**Table 2:** Median and average performance and team total reward for case I (converged or accurate human capabilities)$^\dagger$ and case II (unconverged or inaccurate human capabilities)$^\dagger$ (perf. = performance).

**Case**	**Method**	**Team Perf.**	**Human Perf.**	**Robot Perf.**	**Team Total Reward**
I	ATTA	80(80, 1.7)	96(96, 1.3)	74(74, 2.4)	47(46, 3.1)
	Random	$${66 (66, 2.5)}^{**}$$	$$74 (76, 2.8)^{**}$$	$$58 (58, 3.5)^{**}$$	$$10 (9, 5.2)^{**}$$
	Tsarouchi *et al.*^9^	79(79, 1.5)	$${72 (71, 3.0)}^{**}$$	$${83 (83, 1.3)}^{**}$$	$${38 (39, 3.7)}^{**}$$
II	ATTA	77(77, 2.6)	95(94, 2.3)	72(72, 2.9)	41(42, 4.3)
	Tsarouchi *et al.*^9^ $$({+0.1})$$	$${79 (80, 1.6)}^ {**}$$	$${67 (67, 3.6)}^ {**}$$	$${88 (88, 1.9)}^ {**}$$	$${38 (39, 4.5)}^ {*}$$

$$^{*} p < 0.05$$. $$^{**} p < 0.01$$.

$^\dagger 10$ Simulations: Median (Average, Standard Deviation).

### ATTA method capabilities belief progression and trust evolution

**Figure 5:**
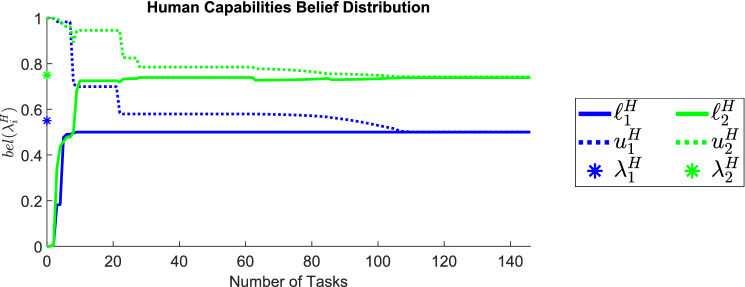
Progression of the human’s capabilities belief distribution. The update in the human’s capabilities belief distribution $bel(\lambda ^H)$ for $(\ell _1^H, u_1^H)$ (blue solid, blue dashed) and $(\ell _2^H, u_2^H)$ (green solid, green dashed) for one sample converged near the human’s actual $\lambda _1^H$ (blue asterisk) and $\lambda _2^H$ (green asterisk) capabilities as task outcomes were observed.

For the ATTA method in case II, the belief over the human’s capabilities is updated as task outcomes from the human are observed. The complete progression of the lower and upper bounds for $bel(\lambda _1^{H})$ and $bel(\lambda _2^{H})$ for this sample are shown in Fig. 5 and the convergence offset is listed in Table 3. The convergence offset between the capabilities belief distribution and the human’s actual capabilities for each capability dimension is computed using Eq. (9), where $\ell _{i,k^H}^H$ and $u_{i,k^H}^H$ are the lower and upper bounds for the human’s capabilities belief distribution for capability dimension $\Lambda _i$ after the human has executed $k^H$ tasks.

9 $$\begin{aligned} convergence \; o\!f\hspace{-0.2em}f\!set_i = |\ell _{i,k^H}^H - \lambda _i^H| + |u_{i,k^H}^H - \lambda _i^H| \end{aligned}$$

As outcomes for tasks allocated to the human were observed, the human’s capabilities belief narrowed and converged near the human’s actual capabilities. When a task was observed as a failure, the upper bound of the capabilities belief decreased to reflect that the human’s actual capabilities were likely to be lower than the failed task requirements. When a task was observed as a success, the lower bound of the capabilities belief increased to reflect that the human’s actual capabilities were likely to be greater than the succeeded task requirements.

**Figure 6:**
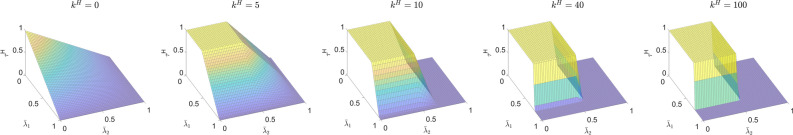
Evolution in human trust. The evolution in human trust across the capability hypercube $\Lambda$ for one sample shows the initial trust distribution ($k^H = 0$) when no task outcomes have been observed to the updated trust distribution ($k^H = 100$) after the capabilities belief converged, which approached a binary value for trust.

The evolution of trust in the human across the capability hypercube $\Lambda$ for this sample is shown in Fig. 6. Since the human’s capabilities were not known initially, trust in the human was built over time. The evolution in human trust across the capability hypercube $\Lambda$ for this sample in Fig. 6 shows how trust was initially distributed, but as the human’s capabilities belief narrowed, trust was more refined. When the human’s capabilities belief converges, trust in the human across the capability hypercube $\Lambda$ approaches a binary value.

## Discussion

Our goal in this research was twofold: first, to develop a human–robot task allocation method that can deal with both unknown agent capabilities and handle novel tasks; and second, to compare this method with existing task allocation methods. We tested our task allocation method in two scenarios: case I, when the human’s capabilities belief distribution has converged to the human’s actual capabilities, and case II, when the human’s capabilities are unknown and initialized as a uniform distribution. Overall, we found the team total reward of our method outperformed other methods for both case I and case II. In this section, we discuss the implications of our findings. The study limitations are discussed in the limitations and future work section.

**Table 3:** Median and average convergence offset after execution of task $$k^H$$ for each capability dimension $$\Lambda _i$$$$^\dagger$$.

**Capability Dimension**	${k^H = 5}$	${k^H = 10}$	${k^H = 20}$	${k^H = 40}$
$\Lambda _1$	0.57(0.60, 0.1)	0.25(0.35, 0.2)	0.14(0.16, 0.1)	0.12(0.12, 0.0)
$\Lambda _2$	0.42(0.41, 0.1)	0.25(0.26, 0.1)	0.13(0.13, 0.0)	0.08(0.08, 0.0)

$^\dagger 10$ Simulations: Median (Average, Standard Deviation).

One contribution of our ATTA method is that agent capabilities are not required to be known in advance but can be learned using stochastic task outcomes. The progression of the human’s capabilities belief distribution for this sample in Fig. 5 and the convergence offset after $k^H$ tasks were executed by the human in Table 3 show how the lower and upper bounds converged near the human’s actual capabilities, proving the effectiveness of our method in determining an agent’s capabilities when they were initially unknown. When a trustee’s capability belief for a capability dimension converges, it represents the point where tasks above were mostly observed as failures and tasks below were mostly observed as successes when executed by the trustee. The capabilities belief started to narrow after observing just a few tasks executed by the human. As the number of tasks to narrow the human’s capabilities belief distribution was relatively low, this can be representative of actual human–robot teams that can quickly determine an agent’s capabilities after observing just a few tasks. While it may take a greater number of tasks to achieve exact convergence between the lower and upper bounds of an agent’s capabilities belief distribution, the agent does not need to execute a long list of tasks immediately. Regardless of when a task is executed, the robot will use the procedure outlined in Eqs. (3) and (4) to refine its belief in an agent’s capabilities.

Despite decreasing convergence offset in the human’s capabilities belief, the progression of the lower and upper bounds reveal an opportunity to make our ATTA method more robust to prevent incorrect convergence as the lower and upper bounds can change quickly after a task outcome. If the human were to fail on a task well below the human’s actual capabilities early on, the capabilities belief may quickly converge below the human’s actual capabilities. In the future, tasks that the human is capable of can yield a low trust due to the incorrectly converged capabilities belief, and may instead get allocated to a less optimal agent. If tasks are not being allocated to the human, our method cannot accurately learn the human’s capabilities. Hence, both the order of task requirements and the level of task requirements can influence the update of the capabilities belief distribution.

A second contribution of our ATTA method is that both existing and novel tasks can be allocated. In our artificial trust model, trust is predicted for any task using the task requirements and the belief in agent capabilities. After trust in an agent to successfully execute the existing or novel task is predicted, the ATTA method allocates the task to an agent on the team. While human–robot teams may encounter many of the same tasks, they may also be faced with novel tasks, especially in dynamic environments (e.g., office, military). It may be impossible to prepare for or predict these tasks ahead of time. Yet, these tasks may be critical and our ATTA method can allocate them.

Although we have simulated our ATTA method with a team composed of one human and one robot, our ATTA method scales to heterogeneous teams with multiple humans and multiple robots by applying the same task allocation method and capabilities belief update procedure to each agent on the team. Trust will be computed for each agent using the capabilities belief distribution for that agent using Eqs. (1) and (2). The capabilities belief distribution for an agent will update after that agent executes a task, and the robot can learn each agent’s capabilities using the same update procedure given in Eqs. (3) and (4). For small enough teams with plenty of tasks to allocate, our ATTA method is expected to work well. To address convergence concerns for larger teams with fewer tasks to allocate, a better initial capabilities estimate for each agent on the team can reduce the number of tasks that need to be executed to achieve a narrow capabilities belief distribution. In addition, the convergence rate can be affected by the level of task requirements and the ordering of the tasks. We do not envision significant calculation complexity issues when allocating tasks for larger teams. Only basic mathematical operations are performed to allocate a task and the calculations to update an agent’s capabilities belief distribution will only be performed once for the agent that executed the task regardless of the size of the team.

## Limitations and future work

Our ATTA method extends existing human–robot task allocation methods, but under some limitations. First, our trust estimate is computed only along the capability dimension of trust, but trust is a function of multiple dimensions. Second, we assume that task requirements are embedded with the incoming task and fall within existing capability dimensions. Like other methods, we cannot handle a task that is entirely outside of the capability hypercube. Third, an agent’s cost and the task reward may depend on other factors, such as the number and complexity of tasks that the agent has already executed or task urgency. Having the human cost depend on other factors can indicate an increased cost due to human fatigue and workload, which can influence the allocation of future tasks. In practice, it may be easier to quantify task requirements, task reward, and agent cost relative to another task instead of absolutely. Fourth, when a task is observed as a failure, we cannot yet determine which capability dimension(s) are responsible for the failure without knowledge of task outcomes from other tasks. Due to this, a capability belief could take longer to converge or converge incorrectly. Fifth, the ATTA method assumes that the time duration to execute each task is the same across agents. If agents take different amounts of time to execute a task, this should be considered so incoming tasks are executed quickly and efficiently. Finally, while the simulation results show potential for using ATTA in real human–robot teams, differences may emerge when used in practice. However, our framework is flexible enough to consider a new trust model, and other agent cost and task reward functions.

Currently, both human and robotic agents have the same mathematical representation, albeit with different parameters. In the future, we plan to incorporate both capability and capacity to capture how agent characteristics can change over time and in different situations^31,72^. Capacity is envisioned to include general elements known to influence performance, such as fatigue or workload for a human. Capability and capacity capture different factors that can influence trust. An agent can be capable of executing a task but not be in the best mental state to do so, or an agent can be mentally available but not have the necessary capabilities to complete a task. Including fatigue or workload can result in a more realistic representation for humans, which will be different from the mathematical representation for robots. After a numerical value is determined for the human’s capacity, this value can be used to scale down the human’s actual capabilities to reflect the human’s capabilities that are available.

Additionally, we have simulated static human capabilities, although human capabilities can be dynamic in practice. We plan to expand the ATTA method to account for dynamic capabilities (e.g., due to human learning).

Also, we have focused on artificial trust from a robotic agent in this paper; we plan to consider the evolution of human trust for task allocation in the future. A human may develop trust differently than the robot that allocates the tasks. Human trust may depend on subjective biases in addition to task outcomes; this needs to be explored further. Due to this, a human may disagree with the task allocation outcome. When there are disagreements among agents^61^, we plan to explore how agents can negotiate the allocation of tasks and how agent preferences can be considered fairly. Negotiation and consideration of preferences are intended not only to enhance team performance, but also to foster team relationship satisfaction (e.g., see^73^).

## Conclusion

This paper presented a task allocation method based on artificial trust in a heterogeneous human–robot team, where trust is the willingness of the trustor to be vulnerable to the actions of the trustee. Our method allows for the allocation of both existing and novel tasks by comparing task requirements with the belief in agent capabilities, and our method learns a trustee agent’s capabilities over time when they are initially unknown using stochastic task outcomes. Our method outperformed other methods in terms of team total reward. This task allocation method can be used in various settings, but is especially beneficial to human–robot collaborative teams handling a variety of tasks or tasks with scalable complexities.

## Supplementary Information

Supplementary Information.

## Data Availability

All data generated and analyzed, along with corresponding code, results, paper figures, and paper source code are available in a public repository at https://github.com/arshaali/artificial-trust-task-allocation.
