# Supplementary material for: Heterogeneous human–robot task allocation based on artificial trust
Source: Sci Rep. 2022 Sep 12;12:15304. doi: 10.1038/s41598-022-19140-5 (PMC9468009; doi:10.1038/s41598-022-19140-5)
Supplement: Supplementary file 1 — Supplementary Information. [file 41598_2022_19140_MOESM1_ESM.pdf]

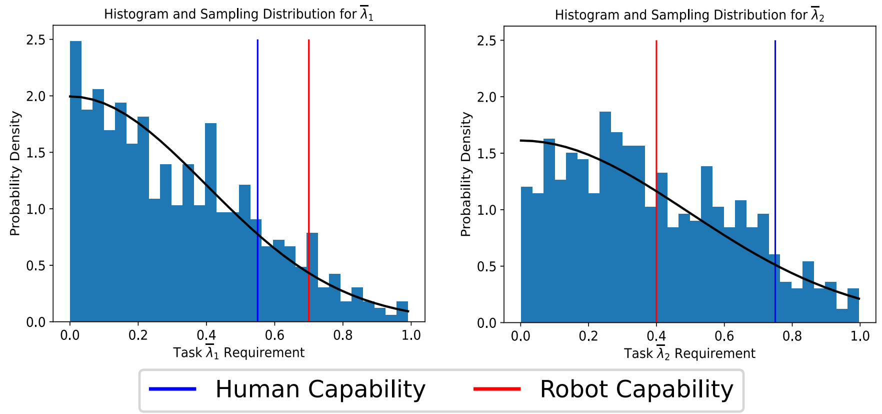


**Figure S1.** Task requirement distributions. The probability density function (black line) and histogram (blue bins) of sampled $\bar{\lambda}_1$ and $\bar{\lambda}_2$ task requirements for one sample of $N = 500$ tasks is shown. The human's capabilities $\lambda_1^H = 0.55$ and $\lambda_2^H = 0.75$ (blue vertical line) and the robot's capabilities $\lambda_1^R = 0.7$ and $\lambda_2^R = 0.4$ (red vertical line) are used to create the probability density functions.

**Algorithm S1** Artificial Trust-Based Task Allocation Method

**procedure** ALLOCATE TASKS($\Gamma, T = \{H, R\}$)
    $bel(\lambda_i^a) \leftarrow \mathscr{U}(\ell_i^a = 0, u_i^a = 1)$ ▷ *initialize uniform capabilities belief distribution*
    **for** $\gamma$ in $\Gamma$ **do**
        ALLOCATE($\gamma, T = \{H, R\}$) ▷ *allocate the task to one agent on the team*
        OBSERVE OUTCOME($\gamma, T = \{H, R\}$) ▷ *observe success or failure of the task*
        **if** $H$ *executed* $\gamma$ **then** ▷ *human executed the task*
            UPDATE CAPABILITIES($bel(\lambda^H)$) ▷ *update human's capabilities belief distribution*
        **end if**
    **end for**
**end procedure**

**procedure** ALLOCATE($\gamma, T = \{H, R\}$)
    $\bar{\lambda} \leftarrow \gamma$ ▷ *task requirements from the task*
    $\tau_\gamma^H \leftarrow ATM(bel(\lambda^H), \bar{\lambda})$ ▷ *trust from ATM*
    $\tau_\gamma^R \leftarrow ATM(bel(\lambda^R), \bar{\lambda})$
    $r_s \leftarrow f_r(\bar{\lambda})$ ▷ *task reward*
    $c^H \leftarrow f_c(H, \bar{\lambda})$ ▷ *agent costs*
    $c^R \leftarrow f_c(R, \bar{\lambda})$
    $\mathbb{E}_\gamma^H \leftarrow \tau_\gamma^H r_s - c^H$ ▷ *expected total rewards*
    $\mathbb{E}_\gamma^R \leftarrow \tau_\gamma^R r_s - c^R$
    **if** $|\mathbb{E}_\gamma^H - \mathbb{E}_\gamma^R| \leq \alpha$ **then** ▷ *expected total rewards within tolerance*
        **if** $k^H \leq k^R$ **then** ▷ *human has fewer than or equal number of tasks to the robot*
            $H \leftarrow \gamma$ ▷ *allocate task to the human*
        **else if** $k^H > k^R$ **then** ▷ *robot has fewer number of tasks than the human*
            $R \leftarrow \gamma$ ▷ *allocate task to the robot*
        **end if**
    **else if** $|\mathbb{E}_\gamma^H - \mathbb{E}_\gamma^R| > \alpha$ **then** ▷ *expected total rewards outside tolerance*
        **if** $\mathbb{E}_\gamma^H > \mathbb{E}_\gamma^R$ **then** ▷ *human has greater expected total reward than the robot*
            $H \leftarrow \gamma$ ▷ *allocate task to the human*
        **else if** $\mathbb{E}_\gamma^H < \mathbb{E}_\gamma^R$ **then** ▷ *robot has greater expected total reward than the human*
            $R \leftarrow \gamma$ ▷ *allocate task to the robot*
        **end if**
    **end if**
**end procedure**

**procedure** OBSERVE OUTCOME($\gamma, T = \{H, R\}$)
    $x \leftarrow random()$ ▷ *x is a random floating number between 0 and 1*
    **if** $H$ *executed* $\gamma$ **then** ▷ *human executed the task*
        **if** $\bar{\tau}_\gamma^H \geq x$ **then** ▷ *probability of human success on the task is greater than or equal to x*
            $\Omega(H, \gamma, t) \leftarrow 1$ ▷ *assign success*
        **else if** $x > \bar{\tau}_\gamma^H$ **then** ▷ *x is greater than probability of human success on the task*
            $\Omega(H, \gamma, t) \leftarrow 0$ ▷ *assign failure*
        **end if**
    **else if** $R$ *executed* $\gamma$ **then** ▷ *robot executed the task*
        **if** $\bar{\tau}_\gamma^R \geq x$ **then** ▷ *probability of robot success on the task is greater than or equal to x*
            $\Omega(R, \gamma, t) \leftarrow 1$ ▷ *assign success*
        **else if** $x > \bar{\tau}_\gamma^R$ **then** ▷ *x is greater than probability of robot success on the task*
            $\Omega(R, \gamma, t) \leftarrow 0$ ▷ *assign failure*
        **end if**
    **end if**
**end procedure**

**procedure** UPDATE CAPABILITIES($bel(\lambda^H)$)
    $bel(\lambda^H) \leftarrow (\hat{\ell}^H, \hat{u}^H)$ following equations (3) and (4) ▷ *update human's capabilities belief distribution*
**end procedure**
